# ﻿A synopsis of the genus *Pulvinora* Davydov, Yakovcz. & Printzen (Lecanoraceae, Lecanorales)

**DOI:** 10.3897/mycokeys.122.152331

**Published:** 2025-09-04

**Authors:** Edyta Mazur, Lucyna Śliwa

**Affiliations:** 1 W. Szafer Institute of Botany, Polish Academy of Sciences, Lubicz 46, 31–512 Kraków, Poland Polish Academy of Sciences Kraków Poland

**Keywords:** Lichenized fungi, *Lecanora* s.l., multilocus phylogeny, new combinations, taxonomy

## Abstract

This study shows that *Lecanora cavicola* and *L. subcavicola* are closely related to the recently described genus *Pulvinora*. These taxa are nested in a sister position to the known *Pulvinora* spp. in a phylogenetic reconstruction based on three loci: mtSSU, nuITS, and nuLSU. The clade incorporating all the above-mentioned taxa is shown to be monophyletic and strongly supported. Consequently, a broader circumscription of the genus is proposed, along with two new taxonomic combinations: *Pulvinora cavicola* (Creveld) Mazur & Śliwa and *Pulvinora subcavicola* (B.D. Ryan) Mazur & Śliwa. Additionally, based on similarity to other members of *Pulvinora*, a new combination for *Lecanora brandegeei* is made. A key for the identification of all *Pulvinora* species is provided as well.

## ﻿Introduction

*Pulvinora* Davydov, Yakovcz. & Printzen is a lichen genus in the family Lecanoraceae. According to the most recent update of the classification of lichen-forming fungi, the family comprises 26 genera (Lücking et al. 2017). However, there are still genera of lecanoroid lichens, such as *Rhizoplaca* (Arup and Grube 2000), that are recognized as polyphyletic. Furthermore, *Lecanora*, the type genus for Lecanoraceae, is also considered as such (e.g., Medeiros et al. 2021). It also included the *Lecanora pringlei* group, which was treated in detail by Ryan and Nash (1997) and Ryan et al. (2004). In the latter publication, the authors proposed segregation of *Lecanora pringlei* (Tuck.) I.M. Lamb., *L. cavicola* Creveld, and *L. subcavicola* B.D. Ryan from the genus *Lecanora* s.l. – a proposal that was finally made by Davydov et al. (2021a), who established the new genus *Pulvinora*.

The phylogenetic reconstruction of *Pulvinora* and other members of Lecanoraceae by Davydov et al. (2021a) was based on phylogenetic analysis of ITS/5.8S, mtSSU, and nuLSU DNA sequences. In addition to, the material of *Lecanora pringlei* and *Pulvinora stereothallina*Davydov et al. (2021a) included DNA sequences from *L. subcavicola* corresponding to the species description in Ryan et al. (2004) and collected by J. Hollinger, F. Bungartz, and C. Parinello from its locus classicus. The material provided a better understanding of the species’ morphology and chemistry and served as a basis for determining its phylogenetic position within the broader *Lecanora* complex. Additionally, the authors (Davydov et al. 2021a) genetically analyzed material labeled by them as L. cf. subcavicola.

Davydov et al. (2021a) concluded that *L. pringlei* from North America and a new related species from the Altai Mountains, *Pulvinora stereothallina*, represent a new, well-supported genus. *Pulvinora pringlei* and *P. stereothallina* share several characteristic features, including a bullate-squamulose to well-stalked, pulvinate thallus and convex apothecia with an excluded thalline margin but an algal layer present under the hypothecium, and a proper exciple (parathecium) that is well developed or thin. While some of the traits delimiting the genus *Pulvinora* are shared with *Lecanora* species (e.g., *Lecanora*-type asci), the combination of thallus morphology, apothecial anatomy, and secondary chemistry is sufficient to distinguish it from close relatives such as the genus *Frutidella* Kalb in the Lecanoraceae (Davydov et al. 2021a).

Two other taxa, *L. subcavicola* and L. cf. subcavicola, were nested as separate species outside of *Pulvinora*, suggesting that they belong to a distinct evolutionary lineage within the broader Lecanoraceae family. It is worth mentioning that another member of the complex, *L. cavicola*, was not included in the genetic analyses by Davydov et al. (2021a) because the authors were unable to obtain fresh material for DNA extraction. Thus, its relationship with *Pulvinora pringlei*, *P. stereothallina*, and other related taxa remained unresolved. This highlighted the need for future research to close this gap and enable a more complete understanding of the genetic and morphological concept of the latter genus.

In the present study, we indicate that both *Lecanora cavicola* and *L. subcavicola* represent one genetic lineage along with the known *Pulvinora* spp. The clade grouping *L. cavicola*, *L. subcavicola*, *Pulvinora pringlei*, and *P. stereothallina* is monophyletic and strongly supported. Therefore, we propose a broader circumscription of the genus *Pulvinora*, with the proposal of three new taxonomic combinations. Distinguishing characters for all discussed species, including *P. brandegeei*, are summarized in a key for their determination.

## ﻿Materials and methods

### ﻿Morphological and chemical analyses

Fresh material of *Lecanora cavicola* from Bolivia available at the KRAM herbarium was investigated. The morphological characters were examined using a Nikon Eclipse i80 light microscope and applying standard techniques. Cross sections were mounted in water or in a solution containing approximately 25% potassium hydroxide (K). Tissue and spore measurements were performed in water. The presence of granulation and crystals was observed under polarized light (pol), and their solubility was assessed using potassium hydroxide (K) and a 65% nitric acid (N) solution. Thin-layer chromatography (TLC) was used for secondary metabolite detection, following the techniques described by Culberson and Kristinsson (1970) and Orange et al. (2001). Lichen substances were analyzed using solvents A and C.

### ﻿﻿Sequence alignments and phylogenetic analyses

Most lichen sequences used in the study were sourced from Medeiros et al. (2021) and extended by selected ones treated in the paper by Davydov et al. (2021a). Only one unpublished sequence of nuITS for *Lecanora cavicola* was added to the dataset, which has now been deposited in GenBank. DNA extraction, PCR, and sequencing were described in detail by Medeiros et al. (2021). The sequences used for the study and their origin are given in Table 1. Phylogenetic analysis was based on three loci: mtSSU, nuITS, and nuLSU. In total, 84 sequences (33, 35, and 16, respectively), including 41 taxa, were used. The final alignment length was 1699 bp (mtSSU: 583; nuITS: 369; nuLSU: 747).

**Table 1.** Specimen data, GenBank accession numbers of the newly generated sequence (in bold), and sequences obtained from GenBank for the taxa used in the phylogenetic analyses.

Species	Origin	Collection and herbarium	GenBank accesion numbers		
			mtSSU	nuITS	nuLSU
*Glaucomaria rupicola* 1	Bolivia	Flakus 29527 (KRAM)	OL604094	OL604012	OL663876
*G. rupicola* 2	Bolivia	Flakus 17372 (KRAM)	–	–	PP447911
*G. rupicola* 3	Bolivia	Flakus 29512 (KRAM)	OL604104	OL604023	OL663884
*G. rupicola* 4	Turkey/NA	MB 0.01 (ERC)/ AFTOL-ID 4894	–	KX550102	KJ766582
*Lecanora achroa*	Thailand	Papong 6458 (F)	JQ782663	JN943714	JN939502
*L. albella*	Bolivia	Flakus 26372 (KRAM)	OL604088	OL604007	OL663872
*L. allophana* 1	Finland	Malíček 9491 (hb. JM)	KY502416	KY548051	–
*L. allophana* 2	Russia	Malíček 9626 (hb. JM)	KY502421	KY548050	–
*L. argopholis*	Austria	CP 12558 (FR-0220001)	MH520108	MH512978	–
*L. caesiorubella*	USA	Lumbsch 19094a (F)	JQ782666	JN943722	JN939506
*L. coronulans*	Bolivia	Flakus 29216 (KRAM)	OL604139	OL604060	OL663918
*L. farinacea*	Australia	Lumbsch 19971b (F)	JQ782670	JN943726	JN939511
*L. flavidomarginata*	Bolivia	Flakus 28943 (KRAM)	OL604077	OL603996	–
*L. flavopallida*	Australia	Lumbsch 19972d	–	JN943723	JN939516
*L. frustulosa*	NA	Lumbsch19608c (F)	–	MG554664	–
*L. gangaleoides*	USA	Lumbsch 19923a (F)	JQ782676	MG554660	–
*L. helva*	Thailand	Papong 5453 (F)	–	JQ782715	–
*L. kenyana*	Kenya	Krika 1179E (F)	–	JQ900618	–
*L. leprosa*	Thailand	Papong 6735 (F)	JQ782682	JQ782721	–
*L. menthoides*	Bolivia	Flakus 27192 (KRAM)	OL604085	OL604004	OL663869
*L. orientoafricana*	Kenya	Krika 2205 (F)	JQ900617	NR_120113	–
*L. plumosa*	Thailand	Papong 6965 (F)	JQ782690	JQ782726	–
*L. pseudoargentata*	Bolivia	Rodriguez 3893 (KRAM)	OL604129	OL604050	OL663908
L. cf. subcavicola 1	USA	Hollinger 6645 (ALTB)	MW257154	–	–
L. cf. subcavicola 2	USA	Hollinger 15676 (FR)	MW257155	–	–
*L. thorstenii*	Bolivia	Rodriguez 3685 (KRAM)	PP447915	PP447908	–
*L. tropica*	Thailand	Papong 6440 (F)	JQ782699	JN943720	JN939518
*L. ulriki*	Thailand	Papong 6476 (F)	JQ782700	–	–
*L. wilsonii*	Australia	HTL20029e (F)	JQ782703	–	–
*Letharia columbiana*	USA	112416	KT453855	KT453735	–
*Pulvinora cavicola* 1	Bolivia	Flakus 29569 (KRAM)	OL604120	**PV175790**	–
*P. cavicola* 2	Bolivia	Flakus 29567 (KRAM)	OL604119	OL604040	OL663900
*P. cavicola* 3	Bolivia	Flakus 29582 (KRAM)	OL604121	OL604041	OL663901
*P. pringlei 1*	USA	McCune BM29823a	–	KF024740	–
*P. pringlei* 2	USA	McCune BM30116	–	KP729374	–
*P. pringlei* 3	USA	McCune 36799 (OSC & ALTB)	MW257153	MW257114	–
*P. stereothallina* 1	Russia	Davydov 14817 (LE)	MW257159	MW257118	–
*P. stereothallina* 2	Russia	Davydov 14821 (ALTB)	MW257156	MW257113	–
*P. stereothallina* 3	Russia	Davydov 14820 (ALTB)	MW257152	MW257112	–
*P. subcavicola* 1	USA	Bungartz 11795 (FR)	MW257158	–	MW257123
*P. subcavicola* 2	USA	Bungartz 11787 (FR)	MW257157	MW257117	–

Trimming the ends of the newly generated sequences was performed using Geneious Prime 2024.0 software. The NCBI nucleotide database was used in conjunction with BLAST to taxonomically identify the sequences and detect any indications of contamination or misidentification of specimens. Three single-locus alignments were prepared for each locus. The sequences were aligned using the MAFFT online server, explicitly utilizing the G-INS-i option as described by Katoh et al. (2019). Subsequently, all instances of uncertain positions were eliminated using Gblocks v. 0.91b (Castresana 2000; Dereeper et al. 2008) through the website, with settings that allow smaller final blocks, gap positions within the final blocks, and less strict flanking positions. Each alignment for a single locus was then used as input for a maximum likelihood phylogenetic analysis conducted in IQ-TREE 1.6.12 (Chernomor et al. 2016; Nguyen et al. 2015), executed on the CIVIB server (Trifinopoulos et al. 2016) to assess incongruence. The best substitution models were selected for each partition using ModelFinder (Kalyaanamoorthy et al. 2017). The models applied in the phylogenetic analysis were as follows: TIMe+G4 for mtSSU, HKY+F+I+G4 for nuITS, and TN+F+G4 for nuLSU. The concatenated alignment, encompassing all loci and representing the fungal symbiotic partner, was used as input for phylogenetic analysis in IQ-TREE, including UFBoot2 bootstrap analysis, SH-aLRT, and approximate Bayes tests. The input partitioning scheme divided the concatenated alignment based on the individual loci. To evaluate support for each single-locus tree, phylogenetic position reconstructions were performed with 5,000 ultrafast bootstrap pseudo-replicates, following the approach outlined by Hoang et al. (2018). Nodes that attained bootstrap values of ≥80% for the SH-aLRT test, >0.95 for the accurate approximation of branch supports (aBayes), or ≥95% for UFBoot2 were considered to be strongly supported. *Letharia columbiana* was used as an outgroup (Zhao et al. 2016; Medeiros et al. 2021). The resulting tree was visualized using FigTree v.1.4.4.

## ﻿Results and discussion

**Figure 1.**
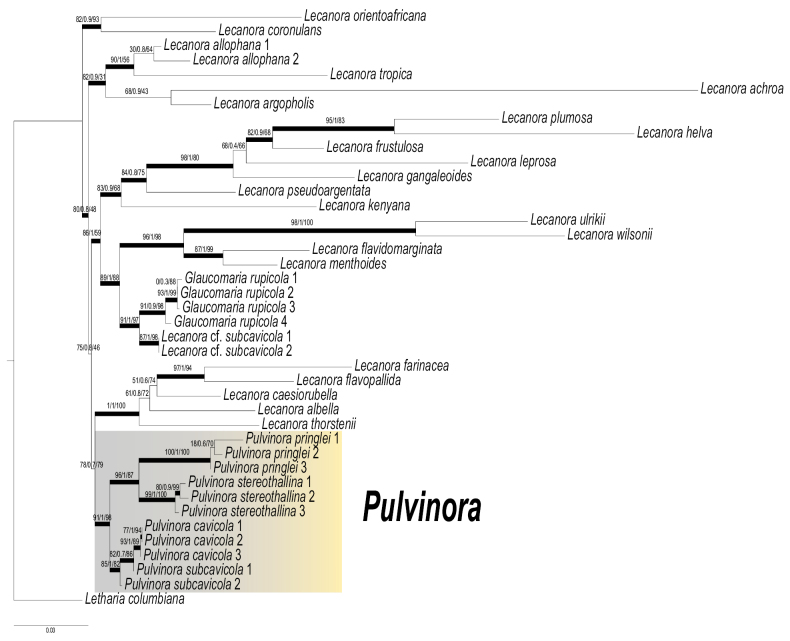
The phylogeny of *Pulvinora* and its related genera based on a concatenated dataset comprising three loci. The concatenated alignment was subjected to SH-aLRT and accurate approximation of branch supports (aBayes) tests, along with UFBoot2 bootstrap analysis utilizing IQ-TREE software. The presented phylogeny is not a consensus tree; branch lengths are optimized values, not averages. Branch lengths represent the number of substitutions per site optimized for the best-fit model and are not averaged across bootstrap replicates. Branches highlighted in bold received bootstrap support of 80% or greater from the SH-aLRT test, more than 0.95 from aBayes branch supports, or 95% or greater from UFBoot2 support.

Most of the presented genetic lineages obtained high statistical support in the phylogenetic reconstruction. Phylogenetic affiliation of the group of lecanoroid lichens selected for study is indicated using a joint tree based on SH-aLRT, accurate approximation of branch supports (aBayes) tests and UFBoot2 bootstrap analysis, and is shown in Fig. 1. Two species, i.e., *Lecanora cavicola* and *L. subcavicola*, form a clade that is sister to the clade of *Pulvinora pringlei* and *P. stereothallina*. All three analyses strongly support this grouping. *Lecanora subcavicola* appears paraphyletic (Fig. 1).

Besides the close phylogenetic relationship, there are also morphological and chemical similarities between *L. cavicola* and *L. subcavicola* and the genus *Pulvinora*. The morphological ones include: (i) the characteristic structure of the thallus, composed of thick areolate-squamulose, verrucose to squamulose, bullate-areolate, or bullate-squamulose units, giving the thallus a crustose to pulvinate appearance (in the latter case, the areoles are narrowed at the base or become indistinctly stalked); areoles/squamules may be plicate or foveolate; and (ii) apothecial features such as an excluded thalline margin but an algal layer present under the hypothecium and a proper exciple (parathecium) that is well developed or thin (cf. Davydov et al. 2021a). Additionally, the species of *L. cavicola* and *L. subcavicola* share the same chemical profile as *Pulvinora*, producing atranorin and alectorialic acid as major secondary lichen metabolites. Therefore, we propose to enlarge the genus by combining *L. cavicola* and *L. subcavicola*, and we propose two relevant taxonomic novelties below. As *L. brandegeei* is also very similar to *Pulvinora* species, we also propose to transfer it to the genus, even though no molecular data are available.

Two specimens treated by Davydov et al. (2021a) as Lecanora cf. subcavicola and placed outside the *Pulvinora* clade are shown to be unrelated to *L. subcavicola* in our phylogeny as well. Their placement on the phylogenetic tree suggests the collection may represent an unknown species closely related to *Glaucomaria rupicola* (L.) P.F. Cannon (Fig. 1).

According to Davydov et al. (2021a), *Pulvinora* is phylogenetically closely related to the genus *Frutidella* and has some similar morphological features, such as clustered convex apothecia. The affiliation of *Frutidella* with the family Lecanoraceae has been strongly supported through phylogenetic reconstruction (Miadlikowska et al. 2014), despite its asci deviating from the typical *Lecanora*-type, being rather *Biatora*-type. *Pulvinora* is also similar to *Miriquidica*, particularly in the shape of apothecia. However, the latter two genera differ in their apothecial anatomy and secondary metabolites. A comprehensive comparison of the genera *Pulvinora*, *Miriquidica*, and *Frutidella* is given by Davydov et al. (2021a).

### ﻿Taxonomy

#### ﻿ Pulvinora brandegeei

Taxon classificationFungiLecanoralesLecanoraceae

(Tuck.) Mazur & Śliwa comb. nov.

5F6FBF6C-D027-5FD1-818F-E933B3056A33

858045

Lecidea brandegeei Tuck. [as ‘brandegei’], Bull. Torrey Bot. Club 10: 21 (1883). Basionym.

##### Type.

U.S.A.‘Colorado • [Chaffee Co.], St. Elmo, 1880, T.S. Brandegee 25’[lectotype FH 00513679! – designated by Davydov & Printzen, The Bryologist 124(2): 251. 2021].

##### Notes.

The species *Lecanora brandegeei* is described in detail and discussed in publications by Lamb (1939), Ryan et al. (2004), McCune (2017), and recently also by Davydov et al. (2021a, b). McCune (2017) specifically accepts it to be synonymous with *L. pringlei*, which is substantiated by Tuckerman (1883), who also indicated a close affinity between the two species. Ryan et al. (2004) proposed *L. brandegeei* as a subspecies of *L. pringlei*, whereas Davydov et al. (2021a, b) suggested a new combination, bringing the taxon to the species level again. The two species morphologically differ from each other in their thallus morphology. The thallus of *L. pringlei* is quite stalked and pseudopodetioid in nature, having cushion-like habits, whereas the thallus of *L. brandegeei* is squamulose with slightly swollen squamules. A broader morphological concept of the genus *Pulvinora* by inclusion of *P. cavicola* and *P. subcavicola* also allows *L. brandegeei* to be a member of the genus. Moreover, the chemistry of the latter species shows similarity to *P. pringlei*, as it produces atranorin, alectorialic and psoromic acids. Therefore, even with no molecular support, the inclusion of *L. brandegeei* appeared fully reasonable.

#### ﻿ Pulvinora cavicola

Taxon classificationFungiLecanoralesLecanoraceae

(Creveld) Mazur & Śliwa comb. nov.

54729FC3-A792-59AA-B795-3A82255ACD26

857746

Fig. 2

Lecanora cavicola Creveld, Bibliotheca Lichenologica 17: 273 (1981). Basionym.

##### Type.

Norway • ‘SE slope Vesl. Nystuguhӧ; Sӧr Trӧndelag, 62°18'NBr, 9°34'OL, d.d. juli 1976, Leg. M. Arnolds-Creveld, Det.: MAC’ (isotype GZU 000291029!).

##### Description.

Thallus creamy to pale greenish, inconspicuous and strongly reduced, or thick, verruculose, areolate to squamulose. Areoles convex, bullate, and irregular, often with craterous, creamy, or light-orange soredia dispersed on the whole thallus, 0.4–3 μm in diam. Prothallus present, black and film-like or pale blue, green, or white and filamentous. Apothecia absent or scarce, sessile, ca. 0.3–0.8 mm in diam. Apothecium margin lecanorine, continuous, and soon excluded. Disc convex, sandy in color. Amphithecium, measured in the middle of thalline margin, is 115 μm wide. Algae are present and scarce. Amphithecial cortex gelatinous, ca. 20 μm. Parathecium hyaline. Epihymenium olive-brown, HCl–, K+ (discolored), and N+ (red-orange). Hymenium hyaline, ca. 70 μm high. Subhymenium not visible. Hypothecium hyaline, up to 125 μm high. Any granules and crystals are not visible in the ascomata. Paraphyses are simple, slender, and unthickened—the apical and basal parts measure 2.5 μm. Asci clavate, 8-spored. Spores hyaline, simple, broadly ellipsoid; 9.0–(9.5)–10.0 × 5.0–(5.5)–6.0 μm (N = 40), L/W ratio = 1.8 μm. Pycnidia not observed.

**Figure 2.**
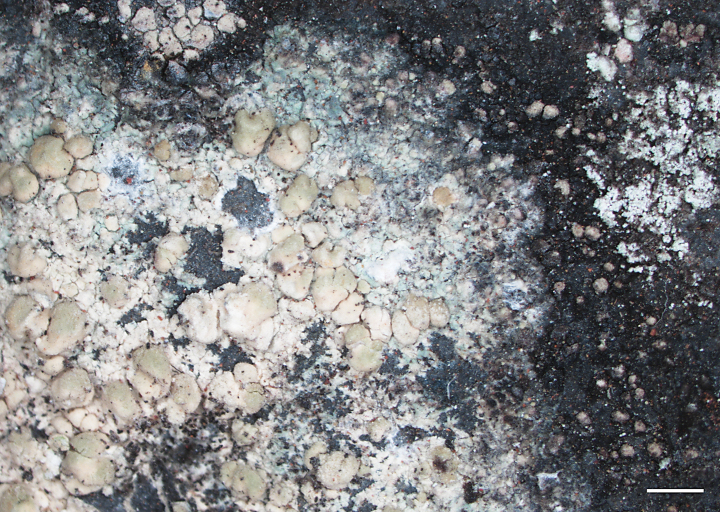
Thallus outline, center, and apothecia of *Pulvinora cavicola* (Creveld) Mazur & Śliwa (KRAM, AF29582). Scale bar: 1 cm.

##### Chemistry.

Atranorin, alectorialic acid, and thamnolic acid (for specimens *A. Flakus 29582* and *29567*) or, perlatolic, alectorialic, and thamnolic acids present (*A. Flakus 29569*).

##### Specimens examined.

Bolivia • Dept. La Paz, Prov. Bautista Saavedra, Área Natural de Manejo Integrado Nacional APOLOBAMBA – protected area, road Pelechuco to Charazani, close to Apacheta, elev. 4262 m, 14°48'27"S, 69°08'05"W, 2017, *A. Flakus 29582*, *29567, 29569* (KRAM, LPB).

##### Exsiccate seen.

Weber, *Lich*. *Exsicc*. *COLO* 135, identified by Poelt, *pro parte* (as *Lecanora pringlei*; cf. Weber 2008) in KRAM.

Reference materials have also been seen from Poland, Tatra Mts., and Bolivia (KRAM; for specimen list see Flakus 2014 and Śliwa et al. 2012).

##### Notes.

Detailed descriptions of the species (under the name *L. cavicola*) are also presented in Poelt and Leuckert (1984) and Ryan et al. (2004). The authors have consistently stated that it occupies an isolated position within *Lecanora*, which was finally confirmed in this study by demonstrating *L. cavicola* placement in the recently established genus *Pulvinora*. The species was described from Norway at the end of the previous century (Creveld 1981) and subsequently reported from various countries in Europe and from other continents. The species appears to be unique among crustose lecanoroid lichens, and its identity can be easily recognized by the pinkish soralia (reflecting alectorialic acid content) in stored herbarium material. The known variation of the species’ secondary chemistry and its geographical range was discussed by Śliwa et al. (2012) while reporting it for the first time from South America, where it was discovered in a few Bolivian provinces. The species produces atranorin and alectorialic acid and, according to the original description, also thamnolic acid (Creveld 1981). However, chemotypes lacking thamnolic acid are commonly reported, and on the other hand, protocetraric acid complex was recorded in addition to atranorin and alectorialic acid (Śliwa et al. 2012). *Pulvinora cavicola* occurs on hard siliceous rocks in alpine areas. Although it is widespread, it is a rare species (Ryan et al. 2004).

#### ﻿ Pulvinora subcavicola

Taxon classificationFungiLecanoralesLecanoraceae

(B.D. Ryan) Mazur & Śliwa comb. nov.

3F200BEA-4F6A-5738-A937-7C01F2FBC4B0

857747

Lecanora subcavicola B.D. Ryan., in Nash et al. (eds). Lich. Fl. Sonoran 2: 269 (2004). Basionym.

##### Notes.

A detailed description of the species (under the name *L. subcavicola*) is provided by Ryan et al. (2004). In morphology and secondary metabolite content (atranorin and alectorialic acid present), it closely resembles *P. cavicola*, except that *P. subcavicola* does not produce vegetative propagules. There is no obvious molecular distinction between the two species on our phylogenetic tree, and more extended sampling in the future will perhaps resolve this issue. However, we were able to indicate differences between *P. cavicola* and *P. subcavicola*, based on the published data – such as the color of the epihymenium: olive-brown (*P. cavicola*) vs. brown-black (*P. subcavicola*); lack (*P. cavicola*) vs. presence (*P. subcavicola*) of fine granules in the epihymenium; and differing chemistry, which is more diverse in the case of *P. cavicola*. A thorough discussion of the latter species’ similarities, differences, and correlations with *P. brandegeei* is available in Ryan et al. (2004) and Davydov et al. (2021a).

### ﻿Key to the species of *Pulvinora*

**Table:** 

1	Thallus pulvinate, bullate-squamulose to distinctly stalked or pseudopodetioid; apothecia protruding above the thallus to stipitate, rarely broadly attached or immersed; parathecium indistinct	**2**
–	Thallus crustose, verrucose, areolate to squamulose, the latter often narrowed at the base; apothecia adnate to sessile, rarely becoming constricted at the base; parathecium indistinct or well developed	**3**
2	Thalline squamules convex; apothecia single; ascospores 7.5−10.0 × 3.0−4.5 µm; atranorin, alectorialic acid, psoromic acid, ±norstictic acid	***P. pringlei***
–	Thalline squamules plane to concave; apothecia coalescing into clusters; ascospores 10.0−15.0 × 3.5−5.0 µm; atranorin and stictic acid complex	***P. stereothallina***
3	Thallus esorediate	**4**
–	Thallus sorediate; ascospores 6.5−13.0 × 5.0−7.0 µm; atranorin, alectorialic acid, ±thamnolic acid, ±perlatolic acid, ±protocetraric acid complex	***P. cavicola***
4	Thallus bullate-areolate, to ca. 3 cm across, 1−2 mm thick; epihymenium brown-black, K−, N+ red, inspersed with fine granules; ascospores 5.0−12.0 × 4.0−7.5 µm; atranorin, alectorialic acid, and unknowns	***P. subcavicola***
–	Thallus areolate-squamulose, 1.5 cm across, 5 mm thick; epihymenium blue-black, K+ blue-green, N+ red-violet, without granules; ascospores 6.0−11.0 × 4.5−5.5 µm; atranorin, alectorialic acid, psoromic acid	***P. brandegeei***

## ﻿Conclusion

The research presented here aimed to bring the genus *Pulvinora* closer to its full circumscription. This was made possible by including the previously missing species *L. cavicola* in the phylogenetic framework. The three-locus analyses showed a close correlation between both *L. cavicola* and *L. subcavicola* and *Pulvinora*, resulting in the newly proposed taxonomic combinations and a broader definition of the latter genus. Consequently, the morphologically similar *L. brandegeei* was also proposed for transfer to the genus. The taxonomic status of L. cf. subcavicola remains unknown – however, its affinity with the genus *Glaucomaria* was clearly indicated.

## Supplementary Material

XML Treatment for Pulvinora brandegeei

XML Treatment for Pulvinora cavicola

XML Treatment for Pulvinora subcavicola

## ﻿Additional information

### Use of AI

No use of AI was reported.

### Author ORCIDs

Edyta Mazur https://orcid.org/0000-0003-2628-5473

Lucyna Śliwa https://orcid.org/0000-0002-7662-1415

### Data availability

All of the data that support the findings of this study are available in the main text.
